# Association of functional polymorphisms in *CYP19A1 *with aromatase inhibitor associated arthralgia in breast cancer survivors

**DOI:** 10.1186/bcr2813

**Published:** 2011-01-20

**Authors:** Jun J Mao, H Irene Su, Rui Feng, Michelle L Donelson, Richard Aplenc, Timothy R Rebbeck, Frank Stanczyk, Angela DeMichele

**Affiliations:** 1 Department of Family Medicine and Community Health, University of Pennsylvania School of Medicine, 3400 Spruce Street/2 Gates, Philadelphia, PA 19104, USA; 2 Abramson Cancer Center, University of Pennsylvania School of Medicine, 3400 Spruce Street/2 Gates, Philadelphia, PA 19104, USA; 3 Department of Reproductive Medicine, University of California School of Medicine, San Diego, 3855 Health Sciences Drive, Dept. 0901, La Jolla, CA 92093-0901, USA; 4 Center for Clinical Epidemiology and Biostatistics, University of Pennsylvania School of Medicine, 423 Guardian Drive, Philadelphia, PA 19104, USA; 5 Department of Hematology/Oncology, Children's Hospital of Philadelphia, 4018 CTRB, 3501 Civic Center Boulevard, Philadelphia PA 19104, USA; 6 Department of Medicine, Division of Hematology/Oncology, University of Pennsylvania School of Medicine, Perelman Center, 3rd Floor, 3400 Civic Center Blvd., Philadelphia, PA 19104, USA

## Abstract

**Introduction:**

Aromatase inhibitor-associated arthralgia (AIAA) is a common and often debilitating symptom in breast cancer survivors. Since joint symptoms have been related to estrogen deprivation through the menopausal transition, we hypothesized that genetic polymorphisms in *CYP19A1*, the final enzyme in estrogen synthesis, may be associated with the occurrence of AIAA.

**Methods:**

We performed a cross-sectional study of postmenopausal women with stage 0 to III breast cancer receiving adjuvant aromatase inhibitor (AI) therapy. Patient-reported AIAA was the primary outcome. DNA was genotyped for candidate *CYP19A1 *polymorphisms. Serum estrogen levels were evaluated by radioimmunoassay. Multivariate analyses were performed to examine associations between AIAA and genetic variants controlling for possible confounders.

**Results:**

Among 390 Caucasian participants, 50.8% reported AIAA. Women carrying at least one 8-repeat allele had lower odds of AIAA (adjusted odds ratio (AOR) 0.41, 95% confidence interval (CI) 0.21 to 0.79, *P *= 0.008) after adjusting for demographic and clinical covariates. Estradiol and estrone were detectable in 47% and 86% of subjects on AIs, respectively. Although these post-AI levels were associated with multiple genotypes, they were not associated with AIAA. In multivariate analyses, women with more recent transition into menopause (less than five years) were significantly more likely to report AIAA than those greater than ten years post-menopause (AOR 3.31, 95% CI 1.72 to 6.39, *P *< 0.001).

**Conclusions:**

Functional polymorphism in *CYP19A1 *and time since menopause are associated with patient-reported AIAA, supporting the hypothesis that the host hormonal environment contributes to the pathophysiology of AAIA. Prospective investigation is needed to further delineate relationships between host genetics, changing estrogen levels and AIAA.

## Introduction

Joint pain, or arthralgia, has emerged as a major symptom in breast cancer survivors on aromatase inhibitors (AIs) for adjuvant hormonal therapy [1,2]. In clinical settings outside of therapeutic trials, close to half of patients on AIs attribute arthralgia to this class of medication [3,4]. AI-associated arthralgia (AIAA) results not only in decreased function [5], but also in premature discontinuation and sub-optimal adherence [6]. Thus, this symptom has the potential to impair both quality of life and drug effectiveness.

Although the pathophysiology of AIAA remains unclear, estrogen suppression is hypothesized to play an important role, since AIs block the final step in estradiol and estrone synthesis [7]. Natural menopause has been associated with increased joint aches and stiffness; symptoms are most prominent during the late menopausal transition when marked falls in circulating estrogen levels occur [8]. Among breast cancer survivors, clinical risk factors associated with AIAA include shorter time since menopause [3] and chemotherapy exposure [4], which further diminishes residual ovarian estrogen production. Thus, estrogen suppression, the main effect of AI exposure, appears linked to arthralgia.

Aromatase enzyme, encoded by *CYP19A1 *and inhibited by AIs, contains common genetic variants that have been associated with circulating estrogen levels in postmenopausal women [9-12]. In particular, intron 4 contains a tetranucleotide repeat polymorphism (TTTA)_*n *= 7-13 _associated with estrogen levels. Postmenopausal women who carry at least one 7-repeat allele (TTTA7) have been found to have lower circulating estrone and estradiol levels; those who carry at least one 8 -repeat allele (TTTA8) have been noted to have higher estrone and estradiol levels, compared to those with all other repeat lengths.

Since polymorphisms in *CYP19A1 *impact estrogen levels, we hypothesized that the presence of functional polymorphisms in this gene would be associated with AIAA among postmenopausal breast cancer survivors on AI therapy. To test this hypothesis, we performed a cross-sectional study of postmenopausal women taking AIs to evaluate whether these polymorphisms were associated with patient-reported occurrence of AIAA. Additionally, we tested the feasibility of measuring estradiol and estrone levels in postmenopausal women on AIs and explored their association with candidate genotypes and AAIA.

## Materials and methods

### Study design and patient population

The Wellness After Breast Cancer (WABC) Study is a cross-sectional study conducted between March 2008 and July 2009 at the Rowan Breast Cancer Center of the Abramson Cancer Center of the University of Pennsylvania (Philadelphia, PA, USA). Eligibility criteria included postmenopausal status (≥12 months of amenorrhea), history of histologically-confirmed hormone receptor-positive breast cancer, AJCC stages 0 to III, and exposure to a third-generation aromatase inhibitor (anastrozole, letrozole, or exemestane). Additional eligibility criteria included completion of all chemotherapy and/or radiotherapy at least one month prior to enrollment, approval of the patient's primary oncologist, and ability to provide informed consent. Research assistants screened medical records and approached potential patients for enrollment at their regular follow-up appointments. After informed consent was obtained, each participant completed a self-administered survey. Peripheral blood was collected; whole blood and serum samples were banked at -80°C for genetic and biomarker analysis, respectively. The study was approved by the Institutional Review Board of the University of Pennsylvania.

### Outcome measurement

We first asked whether participants experienced ongoing joint pain, or arthralgia. Because arthralgia in a postmenopausal female population can be multi-factorial, we then specifically asked participants to attribute their current arthralgia to several factors included aging, AIs, and other medical conditions and medications. As in our prior work, patients who reported AI as a current cause of arthralgia were defined as having AAIA [3]. We also asked those who stopped AIs for discontinuation reasons. Because AIAA is an important cause of premature discontinuation of therapy [13], those who reported stopping AIs because of joint pain or musculoskeletal problems were also classified as having AIAA.

Multiple covariates were ascertained. Patients self-reported demographic variables included age, race/ethnicity, education status, height, weight, date of last menstrual period (LMP) and reasons for menopause (natural, surgery, chemotherapy, hormonal therapy, and other). Clinical variables such as cancer stage, prior chemotherapy, current aromatase inhibitor use, and time since AI start were obtained via chart abstraction and verified by an oncologist (AD) for quality control.

### Polymorphism selection and genotyping

Detailed literature and National Center for Biotechnology Information single nucleotide polymorphism database searches were performed to identify variants in *CYP19A1 *that (1) had a functional impact on gene expression, (2) were associated with either estrogen levels, arthralgia or estrogen withdrawal symptoms in the literature, and (3) had minor allele frequencies of >10%. Five variants in *CYP19A1 *met these criteria. Variants in *CYP19A1 *(IVS1 G/A (rs749292), IVS2 C/A (rs727479), 3'UTR T/C (rs10046), IVS4 -/TCT (rs11575899), TTTA_n _(rs60271534)) are associated with estrogen levels [9,10,14] and/or hot flashes, another symptom related to estrogen withdrawal [12].

Genomic DNA was extracted from stored blood samples using the Qiagen QiaAmp 96 DNA Blood Kit (Valencia, CA, USA). Laboratory personnel were blinded to all clinical and outcome data. Genotyping for *CYP19A1 *TTTA_n _was performed using site-specific primers for PCR amplification according to Woods *et al. *[12] and Kelberman *et al. *[15] with modifications to PCR conditions followed by direct sequencing. For all other SNPs, genotyping was performed using Applied Biosystems' SNPlex platform (Foster City, CA, USA).

### Estrone and estradiol levels

Serum samples were assayed for estradiol and estrone levels. Samples underwent organic solvent extraction followed by Celite column partition chromatography, followed by radioimmunoassay to quantify estrone and estradiol, the primary circulating hormones in postmenopausal women. Assays were performed in duplicate, and means of duplicates were analyzed. The intra- and inter-assay coefficients of variation were <7% and 12%, respectively. The lower limits of detection for estradiol and estrone were 1.5 pg/mL and 1.7 pg/mL, respectively. Values below detection thresholds were given half of the threshold value in analyses.

### Statistical analysis

Data analysis was performed using STATA 10.0 for Windows (STATA Corporation, College Station, TX, USA). Because genetic heterogeneity, or population stratification, could lead to either spurious association or reduced power, we carried out population-specific analysis and report the results restricted to Caucasian subjects since the number of subjects in other ethnic groups was relatively small. For quality control, Hardy-Weinberg Equilibrium was assessed for each polymorphism using the Pearson chi square test.

First, we examined the association between AIAA and polymorphisms using the χ^2 ^test. Additive, dominant and recessive models were tested separately. The association between AIAA and clinical and demographic variables was tested using the χ^2 ^test or Student's *t*-test, as appropriate. Covariates with *P*-values < 0.2 in bivariable analyses were carried forward to the multivariable model. Because estrogen levels were not normally distributed (even with logarithmic transformation), the Kruskal-Wallis test was used to compare estrogen levels by genotype and AIAA status.

For those genotypes that were found to be significant in the first step, we further determined the gene-outcome association using multivariate logistic regression models, adjusting for covariates including education, length of time since menopause, reasons for menopause, time since start of AI therapy, and chemotherapy regimen. Bonferroni correction was applied because five polymorphisms were tested, and the level of significance was adjusted to *P *< 0.01.

## Results

### Participant characteristics

Of 643 consecutive patients screened, 538 (83.7%) agreed to participate. Among 105 who declined (16.3%), the main reasons were: lack of time to complete survey (*n *= 26, 4%); did not want to participate in research (*n *= 43; 6.7%); and did not want to have an extra blood draw (*n *= 36; 5.6%). Additionally, one subject withdrew consent and nine subjects (1.4%) were further disqualified because they did not meet eligibility criteria upon further review. Of 528 subjects who returned data (82.1%), 501 (77.9%) had both an evaluable survey and a blood sample. Twenty-five subjects were further excluded after chart review revealed metastatic disease (3.9%), resulting in the final sample of 476. This population reflected a 74% response rate among all initially approached subjects and a 78% response rate among those eligible.

For this study, we restricted analysis to the 390 Caucasian subjects (81.9%) out of the entire sample. Among these subjects (Table 1), mean age was 61.6 (standard deviation (SD) 9.9); 206 subjects (56%) had gone through natural menopause. Seventy-six subjects (19.0%) were within five years of menopause, while more than half (53%) reported being greater than 10 years from menopause. Overall, 355 (91%) were currently taking an AI at the time of enrollment, while 9% had discontinued AI therapy by the time of the survey. Among those currently taking an AI, the majority (67.9%) was taking anastrozole. Of the 35 subjects who had discontinued AI treatment, median time since discontinuation (IQR) was 10.2 months (31).

**Table 1:** Demographic characteristics of participants

	N	%
**Total (N, %)**	390	81.9
**Age, years (Mean, SD)**	61.6	9.88
**Educational Level (N, %)**		
High school or less	69	17.7
College or more	321	82.3
**Reasons for menopause (N, %)**		
Natural	206	55.7
Induced	164	44.3
**Years since LMP (N, %)**		
<5	76	19.8
5 to 10	104	27.1
>10	204	53.1
**Body mass index, kg/m2 (Mean, SD)**	26.7	5.6
**Stage (N, %)**		
0 and I	149	38.2
II	200	51.3
III	41	10.5
**Chemotherapy (N, %)**		
None	143	36.7
Chemotherapy, but no Taxane	97	24.9
Chemotherapy included Taxane	150	38.5
**Currently on AIs**	355	91
**Aromatase inhibitors**^**1**^		
Letrozole (Femara)	71	20.0
Anastrozole (Arimidex)	241	67.9
Exemestane (Aromasin)	43	12.1
**Years since start of AI**^**1**^		
<1	114	31.9
1 to 3	111	31.1
>3	132	37.0

Abbreviations: AIs, aromatase inhibitors; LMP, last menstrual period; SD, standard deviation.

^1^Among those who are currently on AIs.

Not all cells add up due to missing variables.

### Patient-reported AI-associated arthralgia

Among the participants, 198 (50.8%) reported joint symptoms attributable to AI or cited arthralgia as reason for their discontinuation of AIs, and were therefore classified as having AIAA. Risk of AIAA was non-statistically higher among those who stopped AIs than among those who were currently on AIs (62.9% vs. 49.6%, *P *= 0.13). Shorter time since menopause (*P *< 0.001), exposure to chemotherapy including taxane (*P *= 0.006), and one to three years since the start of AI exposure (*P *= 0.02) appeared to be associated with greater report of AAIA in univariate analysis (Table 2). Those with AIAA were significantly younger than those without AIAA (59 vs. 65, *P *< 0.001) but had similar body mass index (BMI) (27.3 vs. 27.6, *P *= 0.85).

**Table 2:** Clinical and demographic variables and risk of AAIA

Characteristic	Reported AIAA (*N *= 390)		***P*-value**^**2**^
		
	N	%	
**Educational Level**			0.11
High school or less	29	42.0	
College or more	169	52.7	
**Reasons for menopause**			0.13
Natural	98	47.6	
Induced	91	55.5	
**Years since LMP**			<.001
<5	50	65.8	
5 to 10	63	60.6	
>10	83	40.7	
**Body mass index**			0.62
<25	97	50.5	
25 to 30	68	45.3	
>30	68	49.3	
**Stage**			0.991
0 and I	75	50.4	
II	102	51.0	
III	21	51.2	
**Chemotherapy**			0.006
None	59	41.3	
Chemotherapy, but no Taxane	49	50.2	
Chemotherapy included Taxane	90	60.0	
**Aromatase inhibitors**			0.62
Letrozole (Femara)	33	46.5	
Anastrozole (Arimidex)	119	49.4	
Exemestane (Aromasin)	24	55.8	
**Years since start of AI**^1^			0.02
<1	56	49.1	
1 to 3	67	59.6	
>3	55	41.7	
**Estrogen levels**			
Detectable estradiol	74	47.4	0.84
Detectable estrone	134	85.9	0.73

Abbreviations: AIAA, Aromatase Inhibitor associated Arthralgia; LMP, last menstrual period.

^1^Among those who are currently on AIs.

^2^Chi-square.

### CYP19A1 polymorphisms

All genotyping failure rates were <1.8%. Genotype distributions were in Hardy-Weinberg equilibrium and were consistent with reported reference SNP frequencies. Univariate associations between *CYP19A1 *(aromatase) polymorphisms and study outcomes demonstrated that subjects with at least one (TTTA)_7 _repeat allele had higher risk of AIAA, while subjects with at least one (TTTA)_8 _repeat allele had lower risk of AIAA (Table 3).

**Table 3:** Genotypes and risk of AIAA^1^

Polymorphism	Genotype frequency		AIAA	
		
	Genotype	% (N)	% (N)	***P*-value**^**2**^
	C/C	28.1 (107)	50 (54)	
3' UTR C/T (rs10046)	C/T	47.8 (182)	54 (98)	0.20
	T/T	24.1 (92)	42 (39)	

	G/G	30.8 (118)	54 (63)	
IVS1 A/G (rs749292)	G/A	52.2 (200)	48 (96)	0.57
	A/A	17.0 (65)	54 (35)	

	G/G	13.1 (50)	50 (25)	
IVS2 G/T (rs727479)	G/T	42.7 (163)	52 (84)	0.94
	T/T	44.2 (169)	50 (84)	

	TCT/TCT	44.8 (172)	49 (84)	
IVS4 -/TCT (rs11575899)	TCT/-	41.4 (159)	51 (81)	0.86
	-/-	13.8 (53)	53 (28)	

	No 7 repeat	28.8 (109)	42 (46)	**0.03**
	Any 7 repeat	71.2 (270)	55 (148)	
	
	No 8 repeat	87.1 (330)	53 (176)	**0.03**
	Any 8 repeat	12.9 (49)	37 (18)	
	
(TTTA)n (rs60271534)	No 10 repeat	88.7 (336)	51 (173)	0.75
	Any 10 repeat	11.3 (43)	49 (21)	
	
	No 11 repeat	62.2 (234)	52 (121)	0.92
	Any 11 repeat	37.8 (142)	51 (72)	
	
	No 12 repeat	88.7 (336)	51 (170)	0.63
	Any 12 repeat	11.3 (43)	56 (24)	

Abbreviations: AIAA, Aromatase Inhibitor Associated Arthralgia.

^1^Among Caucasian subjects.

^2^Chi-square.

### Multivariable analysis

Multivariable models examining the association between AIAA and TTTA_7 _and TTTA_8 _genotypes were adjusted for time since LMP, reason for menopause, time since start of AI therapy, education level, and chemotherapy. Age was not included in the model because of co-linearity with time since LMP. Subjects carrying at least one 7-repeat allele had a non-significant 1.7-fold increase in odds of AIAA (*P *= 0.028) after correcting for multiple testing, while subjects carrying an 8-repeat allele had a significantly decreased odds of AIAA (OR 0.41 (0.21, 0.79), *P *= 0.008) (Table 4). In these models, the only significant clinical predictor of AIAA was time since menopause, with those who were less than five years from their LMP at three-fold increased odds of reporting AAIA than those who were more than 10 years from their LMP.

**Table 4:** Multivariable logistic regression analyses: CYP19A variant and AIAA

Risk factors	AOR (95% C.I.)	*P*-value	AOR (95% C.I.)	*P*-value
**Genotype**	***CYP19A *7r**		***CYP19A *8r**	
Non-carrier (Reference)	1	-	1	-
Carrier	1.70 (1.06 to 2.73)	0.028	0.41 (0.21 to 0.79)	**0.008**
**Time since LMP**				
>10 years (Reference)	1	-	1	-
5 to 10 years	2.15 (1.27 to 3.67)	**0.005**	2.14 (1.26 to 3.66)	**0.005**
<5 years	3.28 (1.71 to 6.29)	**<0.001**	3.31 (1.72 to 6.39)	**<0.001**
**Type of menopause**				
Natural (Reference)	1	-	1	-
Induced	0.82 (0.55 to 1.21)	0.32	0.81 (0.55 to 1.20)	0.30
**Years since start of AI**				
<1 year (Reference)	1	-	1	-
1 to 3 years	1.45 (0.82 to 2.58)	0.20	1.48 (0.83 to 2.62)	0.18
>3 years	0.78 (0.45 to 1.33)	0.36	0.77 (0.45 to 1.33)	0.35
**Education**				
High school	1	-	1	-
College and graduate school	1.02 (0.76 to 1.38)	0.87	1.05 (0.78 to 1.43)	0.93
**Chemotherapy**				
None (Reference)	1	-	1	-
Chemotherapy but no taxane	0.97 (0.54 to 1.74)	0.92	1.02 (0.57 to 1.83)	0.85
Chemotherapy included taxane	1.52 (0.89 to 2.61)	0.12	1.61 (0.93 to 2.78)	0.09

Abbreviations: AOR, adjusted odds ratio; 95% CI, 95% confidence interval; LMP, last menstrual period.

### Estrogen levels

**Figure 1:**
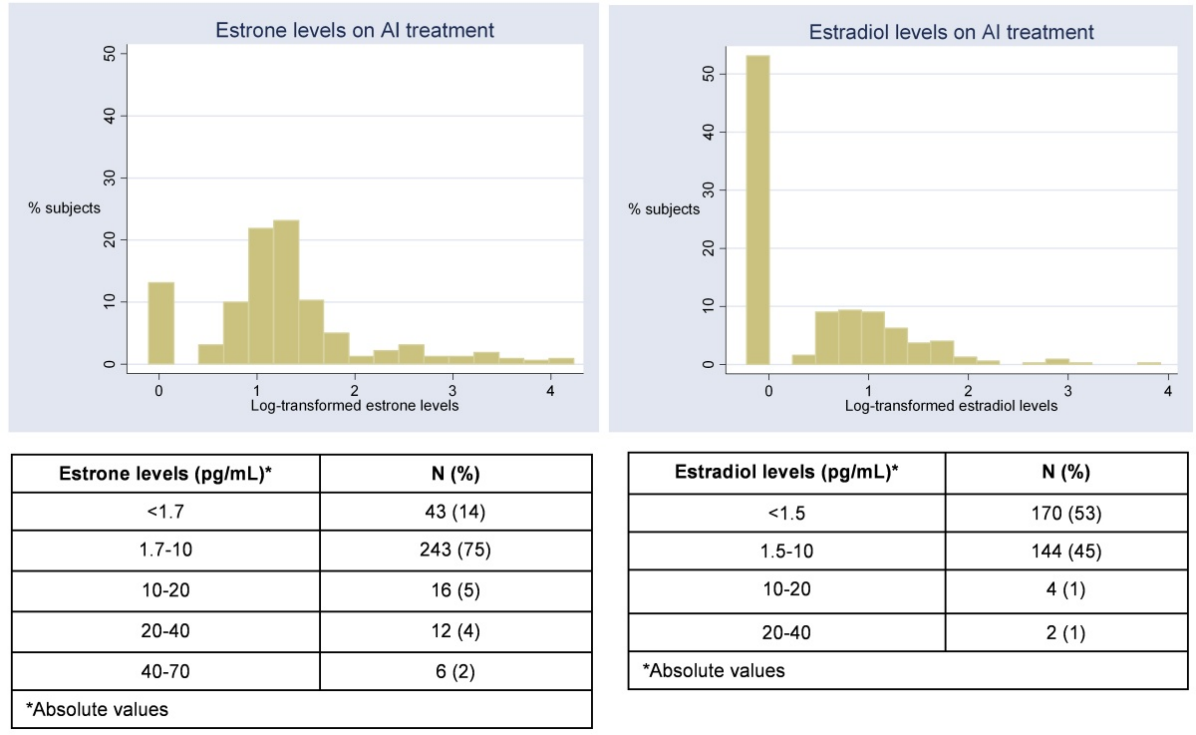
**Estrogen levels among subjects currently on aromatase inhibitors**.

Because AIs are known to result in profound estrogen deprivation, we sought to establish the feasibility of quantifying estrogen levels on women receiving AIs. Of 320 subjects who were currently on AIs and had samples available for estrogen analyses, estradiol was detectable in 150 subjects (46.9%), while estrone was detectable in 277 subjects (86.6%). The median (range) estradiol and estrone levels were <1.5 pg/mL (<1.5, 40.3) and 3.3 pg/mL (<1.7, 60.3), respectively (Figure 1). Estradiol (*P *= 0.88) and estrone (*P *= 0.78) levels were no different by AAIA status.

For subjects who were currently taking AIs, estrone levels were associated with multiple genotypes. At the 3'UTR locus, carriers of the variant T allele had higher estrone levels (median (range)) compared to the homozygous wildtype (3.3 pg/mL (<1.7, 69.1) versus 3.1 pg/mL (<1.7, 32.4), *P *= 0.03). Variant genotypes at the IVS2 polymorphism also had significantly higher estrone levels than the homozygous wildtype genotype (3.3 pg/mL (<1.7, 69.1) versus 2.0 pg/mL (<1.7, 26.7), *P *= 0.01). For the IVS4 polymorphism, the homozygous variant genotype (TCT -/-) was associated with higher estrone levels compared to carriers of the TCT allele (6.3 (<1.7, 18.3) versus 2.9 (<1.7, 32.4), *P *= 0.02). Estradiol levels were not associated with genotypes in this dataset. As expected, estrogen levels were significantly lower in women currently on AIs (*P *< 0.001 for both estradiol and estrone) than those who discontinued AIs. Furthermore, higher estrogen levels were associated with shorter time since menopause (*P *< 0.002 for estrone; *P *= 0.01 for estradiol).

## Discussion

The introduction of AIs as treatment for hormone receptor-positive breast cancer has improved disease free survival for many women; however, arthralgia related to AIs has caused significant symptom distress and may contribute to premature discontinuation among users [16]. We have found that germline polymorphic repeats in *CYP19A1*, the gene encoding aromatase enzyme, are associated with the occurrence of AIAA. Additionally, the study confirmed our prior findings that shorter time since menopause was a significant clinical predictor of report of AAIA [3]. These data provide evidence that the host estrogen environment plays an important role in the occurrence of AIAA and helps explain part of the inter-individual variability in symptom experience among patients taking these agents.

The high rate of AIAA in this study confirms previous reports that AIAA affects almost half of the ambulatory patients who receive AIs [3,4]. These rates are much higher than reported in clinical trial settings (18.6% to 35.6%) [17-20] and may be a result of the differences in patient-reported outcomes versus clinician ascertained toxicity [21]. Emerging research suggests that patient reported toxicity more comprehensively capture the subjective side effects of therapies (that is, pain) on daily experience and have higher concordance with health-related quality of life than clinician ascertained toxicity; therefore, it is more appropriate for the investigation of AIAA [22,23]. Further, only a very small proportion of all cancer patients participate in clinical trials [24]. Thus, it is possible that selection bias in clinical trial participants may also lead to decreased incidence of reported AIAA than the rate in ambulatory settings.

We have identified promising associations between genetic variation in aromatase and AIAA. Carriers of at least one 7-repeat allele in the tetranucleotide repeat polymorphism had non-significant higher risk of AAIA, while carriers of at least one 8-repeat allele had significant lower risk. TTTA_7 _has been associated with lower estrogen levels in postmenopausal women and TTTA_8 _has been associated with higher estrogen levels [14]. These relationships support one possible hypothesis: women with aromatase enzyme polymorphisms associated with lower pre-AI estrogen levels undergo further estrogen depletion with AI exposure, thereby rendering them at higher risk of developing AAIA. Other functional polymorphisms and haplotypes in aromatase that are related to estrogen levels have been described in resequencing projects [14,25], but we did not find a significant association between several of these candidate polymorphisms and AIAA. An alternative hypothesis may be that genetic polymorphisms in aromatase gene may impact the efficacy of aromatase inhibitors and produce varying degrees of estrogen deprivation. Further prospective study combining genotyping, high sensitivity estrogen measurements and AIAA may help test these hypotheses.

Since AIs are known to cause profound estrogen deprivation, measuring estrogen levels in AI users is essential to laying the groundwork for further understanding the relationship between these levels and AIAA. Our assay was highly reliable and extraction methods minimized measurement of other estrogen metabolites and drug metabolites of exemestane. However, we did not find a difference in post-AI estrogen levels by AIAA status. One explanation may be the already low overall levels of these circulating sex hormones observed in the study. Although a highly sensitive RIA method was used, a significant proportion of subjects had estradiol and/or estrone levels below the level of detection, limiting our discrimination. Presently, the assay sensitivity for mass spectrometry (MS) for estradiol is similar to our method (1 to 2 pg/mL). However, as MS technology advances, it will be possible to measure extremely low estrogen levels, in the femotogram range, and improve discrimination [26]. Another explanation is that although we restricted our analysis to those who self-report current use of AIs, it is possible that imperfect adherence to AIs may introduce variability in estrogen levels and thus confound the analysis between estrogen levels and AIAA towards null. Finally, a likely explanation may be that since arthralgia occurs with the exposure of AIs, it is possible that pre-AI estrogen levels or a change in estrogen levels resulting from AI exposure are more critical to the development of AIAA. Our findings of an inverse relationship between time since menopause and estrogen levels and AIAA risk support this hypothesis.

Although estradiol and estrone levels were not associated with AIAA, estrone levels were significantly different among multiple genotypes in the expected direction. Homozygotes for the t allele at 3'UTR, (TCT)+ allele at IVS4 and t allele at IVS2 have been observed to have higher postmenopausal levels of estradiol and estrone [9]. Estrogen levels were no different among other genotypes, including TTTA_n_, a finding that may also be due to the low circulating hormone levels on AIs. In these results, we have shown that the relationship between genotype and estrogen levels remained for some variants even in the setting of AI exposure, and that estrone rather than estradiol levels were measurably different. This suggests the utility of estrone as a potential hormonal biomarker in AI users.

Finally, assessing estradiol and estrone levels in a large cohort of women exposed to AIs has only been done in one recent study [27,28]. Using purification steps to optimize specificity and highly sensitive estradiol and estrone radioimmunoassays, we demonstrated that a large proportion of women had both measurable estrone and estradiol levels. Furthermore, those women who discontinued AI therapy had higher estrogen levels than those who continued. Given the increased concerns about non-adherence in the setting of adjuvant hormonal treatment [29,30], the appropriate measurement of these hormones may help determine how post-AI hormones relate to AI-adherence, breast cancer recurrence, and overall survival in longitudinal studies.

It is important to acknowledge the limitations of the study. Our outcomes were based on patient-report and may be subject to recall bias; however, patient-reported outcomes are considered the gold standard in symptom and pain research. Further the prevalence and risk of AIAA in this study were similar to prior samples by our group and others [3,4], lending credence to this outcome classification. As with any genetic epidemiology study, our results are subject to false-positive discovery; however, our Bonferroni correction strengthens the possibility that this finding is robust. These results warrant further confirmatory analysis in larger and independent cohorts. Third, our cross-sectional study design helps identify a gene-symptom association that will require the prospective investigation in the setting of pre and post AI-exposure and incorporate validated patient-reported outcomes of joint pain as well as high sensitivity estrogen analyses to further determine the role of estrogen deprivation on AIAA. Finally, although our overall sample had significant proportions of non-Caucasian subjects, the samples were too small for meaningful genetic analysis; collaborative studies need to be established to study how genetic polymorphisms affect AIAA in minority populations.

## Conclusions

This is the largest study to date of patient-reported outcomes among ambulatory patients taking AIs for adjuvant breast cancer treatment and the first one to report genetic determinants of AIAA. These data provide preliminary evidence that genes in estrogen synthesis may modify the risk of AIAA. We also established the feasibility of measuring the estrogen levels of ambulatory AI-users which allows for future prospective investigation of the complex relationship among genetic variations, hormonal changes, and AI related arthralgia and breast cancer outcomes. It is conceivable that one day, the combination of genetic and hormonal information may help clinicians and patients decide how best to use AIs to maximize benefits, minimize side effects, and optimize both quality of life and survival in women with breast cancer.

## Abbreviations

AI: aromatase inhibitor; AIAA: aromatase inhibitor associated arthralgia; AOR: adjusted odds ratio; BMI: body mass index; 95% CI: 95% confidence interval; LMP: last menstrual period; RIA: radioimmunoassay.

## Competing interests

The authors declare that they have no competing interests.

## Authors' contributions

JJM, HIS and AD participated in the design of the study and management of study-related data collection. RF performed statistical analyses. MH and RA performed DNA sequencing. TTR assisted data interpretation, and FZS performed estrogen analyses. All authors have read and approved the final manuscript.

## References

[B1] Chlebowski RT Aromatase inhibitor-associated arthralgias J Clin Oncol 2009 27 4932 4934 10.1200/JCO.2009.23.3270 19752332

[B2] Coleman RE Bolten WW Lansdown M Dale S Jackisch C Merkel D Maass N Hadji P Aromatase inhibitor-induced arthralgia: Clinical experience and treatment recommendations Cancer Treat Rev 2008 34 275 282 10.1016/j.ctrv.2007.10.004 18082328

[B3] Mao JJ Stricker C Bruner D Xie S Bowman MA Farrar JT Greene BT DeMichele A Patterns and risk factors associated with aromatase inhibitor-related arthralgia among breast cancer survivors Cancer 2009 115 3631 3639 10.1002/cncr.24419 19517460 PMC3569524

[B4] Crew KD Greenlee H Capodice J Raptis G Brafman L Fuentes D Sierra A Hershman DL Prevalence of joint symptoms in postmenopausal women taking aromatase inhibitors for early-stage breast cancer J Clin Oncol 2007 25 3877 3883 10.1200/JCO.2007.10.7573 17761973

[B5] Morales L Pans S Verschueren K Van Calster B Paridaens R Westhovens R Timmerman D De Smet L Vergote I Christiaens MR Neven P Prospective study to assess short-term intra-articular and tenosynovial changes in the aromatase inhibitor-associated arthralgia syndrome J Clin Oncol 2008 26 3147 3152 10.1200/JCO.2007.15.4005 18474874

[B6] Donnellan PP Douglas SL Cameron DA Leonard RC Aromatase inhibitors and arthralgia J Clin Oncol 2001 19 2767 11352973

[B7] Felson DT Cummings SR Aromatase inhibitors and the syndrome of arthralgias with estrogen deprivation Arthritis Rheum 2005 52 2594 2598 10.1002/art.21364 16142740

[B8] Dugan SA Powell LH Kravitz HM Everson Rose SA Karavolos K Luborsky J Musculoskeletal pain and menopausal status Clin J Pain 2006 22 325 331 10.1097/01.ajp.0000208249.07949.d5 16691084

[B9] Dunning AM Dowsett M Healey CS Tee L Luben RN Folkerd E Novik KL Kelemen L Ogata S Pharoah PD Easton DF Day NE Ponder BA Polymorphisms associated with circulating sex hormone levels in postmenopausal women J Natl Cancer Inst 2004 96 936 945 10.1093/jnci/djh167 15199113

[B10] Haiman CA Hankinson SE Spiegelman D De Vivo I Colditz GA Willett WC Speizer FE Hunter DJ A tetranucleotide repeat polymorphism in CYP19 and breast cancer risk Int J Cancer 2000 87 204 210 10.1002/1097-0215(20000715)87:2<204::AID-IJC8>3.0.CO;2-3 10861475

[B11] Tworoger SS Chubak J Aiello EJ Ulrich CM Atkinson C Potter JD Yasui Y Stapleton PL Lampe JW Farin FM Stanczyk FZ McTiernan A Association of CYP17, CYP19, CYP1B1, and COMT polymorphisms with serum and urinary sex hormone concentrations in postmenopausal women Cancer Epidemiol Biomarkers Prev 2004 13 94 101 10.1158/1055-9965.EPI-03-0026 14744739

[B12] Woods NF Mitchell ES Tao Y Viernes HM Stapleton PL Farin FM Polymorphisms in the estrogen synthesis and metabolism pathways and symptoms during the menopausal transition: observations from the Seattle Midlife Women's Health Study Menopause 2006 13 902 910 10.1097/01.gme.0000227058.70903.9f 16977255

[B13] Fontaine C Meulemans A Huizing M Collen C Kaufman L De Mey J Bourgain C Verfaillie G Lamote J Sacre R Schallier D Neyns B Vermorken J De Greve J Tolerance of adjuvant letrozole outside of clinical trials Breast 2008 17 376 381 10.1016/j.breast.2008.02.006 18455395

[B14] Haiman CA Dossus L Setiawan VW Stram DO Dunning AM Thomas G Thun MJ Albanes D Altshuler D Ardanaz E Boeing H Buring J Burtt N Calle EE Chanock S Clavel-Chapelon F Colditz GA Cox DG Feigelson HS Hankinson SE Hayes RB Henderson BE Hirschhorn JN Hoover R Hunter DJ Kaaks R Kolonel LN Le Marchand L Lenner P Lund E Genetic variation at the CYP19A1 locus predicts circulating estrogen levels but not breast cancer risk in postmenopausal women Cancer Res 2007 67 1893 1897 10.1158/0008-5472.CAN-06-4123 17325027

[B15] Kelberman D Fife M Rockman MV Brull DJ Woo P Humphries SE Analysis of common IL-6 promoter SNP variants and the AnTn tract in humans and primates and effects on plasma IL-6 levels following coronary artery bypass graft surgery Biochim Biophys Acta 2004 1688 160 167 14990346 10.1016/j.bbadis.2003.11.010

[B16] Burstein HJ Winer EP Aromatase inhibitors and arthralgias: a new frontier in symptom management for breast cancer survivors J Clin Oncol 2007 25 3797 3799 10.1200/JCO.2007.11.9529 17761968

[B17] Coates AS Keshaviah A Thurlimann B Mouridsen H Mauriac L Forbes JF Paridaens R Castiglione-Gertsch M Gelber RD Colleoni M Lang I Del Mastro L Smith I Chirgwin J Nogaret JM Pienkowski T Wardley A Jakobsen EH Price KN Goldhirsch A Five years of letrozole compared with tamoxifen as initial adjuvant therapy for postmenopausal women with endocrine-responsive early breast cancer: update of study BIG 1-98 J Clin Oncol 2007 25 486 492 10.1200/JCO.2006.08.8617 17200148

[B18] Coombes RC Kilburn LS Snowdon CF Paridaens R Coleman RE Jones SE Jassem J Van de Velde CJ Delozier T Alvarez I Del Mastro L Ortmann O Diedrich K Coates AS Bajetta E Holmberg SB Dodwell D Mickiewicz E Andersen J Lonning PE Cocconi G Forbes J Castiglione M Stuart N Stewart A Fallowfield LJ Bertelli G Hall E Bogle RG Carpentieri M Survival and safety of exemestane versus tamoxifen after 2-3 years' tamoxifen treatment (Intergroup Exemestane Study): a randomised controlled trial Lancet 2007 369 559 570 10.1016/S0140-6736(07)60200-1 17307102

[B19] Goss PE Ingle JN Martino S Robert NJ Muss HB Piccart MJ Castiglione M Tu D Shepherd LE Pritchard KI Livingston RB Davidson NE Norton L Perez EA Abrams JS Cameron DA Palmer MJ Pater JL Randomized trial of letrozole following tamoxifen as extended adjuvant therapy in receptor-positive breast cancer: updated findings from NCIC CTG MA.17 J Natl Cancer Inst 2005 97 1262 1271 10.1093/jnci/dji250 16145047

[B20] Howell A Cuzick J Baum M Buzdar A Dowsett M Forbes JF Hoctin-Boes G Houghton J Locker GY Tobias JS Results of the ATAC (Arimidex, Tamoxifen, Alone or in Combination) trial after completion of 5 years' adjuvant treatment for breast cancer Lancet 2005 365 60 62 10.1016/S0140-6736(05)74803-0 15639680

[B21] Basch E Iasonos A McDonough T Barz A Culkin A Kris MG Scher HI Schrag D Patient versus clinician symptom reporting using the National Cancer Institute Common Terminology Criteria for Adverse Events: results of a questionnaire-based study Lancet Oncol 2006 7 903 909 10.1016/S1470-2045(06)70910-X 17081915

[B22] Basch E Jia X Heller G Barz A Sit L Fruscione M Appawu M Iasonos A Atkinson T Goldfarb S Culkin A Kris MG Schrag D Adverse symptom event reporting by patients vs clinicians: relationships with clinical outcomes J Natl Cancer Inst 2009 101 1624 1632 10.1093/jnci/djp386 19920223 PMC2786917

[B23] Neben-Wittich MA Atherton PJ Schwartz DJ Sloan JA Griffin PC Deming RL Anders JC Loprinzi CL Burger KN Martenson JA Miller RC Comparison of provider-assessed and patient-reported outcome measures of acute skin toxicity during a phase iii trial of mometasone cream versus placebo during breast radiotherapy: The North Central Cancer Treatment Group (N06C4) Int J Radiat Oncol Biol Phys 2010 in press 20888137 10.1016/j.ijrobp.2010.05.065 PMC3365545

[B24] Murthy VH Krumholz HM Gross CP Participation in cancer clinical trials: race-, sex-, and age-based disparities JAMA 2004 291 2720 2726 10.1001/jama.291.22.2720 15187053

[B25] Ma CX Adjei AA Salavaggione OE Coronel J Pelleymounter L Wang L Eckloff BW Schaid D Wieben ED Adjei AA Weinshilboum RM Human aromatase: gene resequencing and functional genomics Cancer Res 2005 65 11071 11082 10.1158/0008-5472.CAN-05-1218 16322257

[B26] Stanczyk FZ Clarke NJ Advantages and challenges of mass spectrometry assays for steroid hormones J Steroid Biochem Mol Biol 2010 121 491 495 10.1016/j.jsbmb.2010.05.001 20470886

[B27] Ingle JN Buzdar AU Schaid DJ Goetz MP Batzler A Robson ME Northfelt DW Olson JE Perez EA Desta Z Weintraub RA Williard CV Flockhart DA Weinshilboum RM Variation in anastrozole metabolism and pharmacodynamics in women with early breast cancer Cancer Res 2010 70 3278 3286 10.1158/0008-5472.CAN-09-3024 20354183 PMC2855746

[B28] Dixon JM Renshaw L Young O Murray J Macaskill EJ McHugh M Folkerd E Cameron DA A'Hern RP Dowsett M Letrozole suppresses plasma estradiol and estrone sulphate more completely than anastrozole in postmenopausal women with breast cancer J Clin Oncol 2008 26 1671 1676 10.1200/JCO.2007.13.9279 18375896

[B29] Partridge AH Wang PS Winer EP Avorn J Nonadherence to adjuvant tamoxifen therapy in women with primary breast cancer Journal of Clinical Oncology 2003 21 602 606 10.1200/JCO.2003.07.071 12586795

[B30] Hershman DL Kushi LH Shao T Buono D Kershenbaum A Tsai WY Fehrenbacher L Gomez SL Miles S Neugut AI Early discontinuation and nonadherence to adjuvant hormonal therapy in a cohort of 8,769 early-stage breast cancer patients J Clin Oncol 2010 28 4120 4128 10.1200/JCO.2009.25.9655 20585090 PMC2953970

